# On-demand intermittent beclomethasone is effective for mild asthma in Brazil

**DOI:** 10.1186/s13601-018-0192-0

**Published:** 2018-03-05

**Authors:** Paulo Camargos, Alessandra Affonso, Geralda Calazans, Lidiana Ramalho, Marisa L. Ribeiro, Nulma Jentzsch, Simone Senna, Renato T. Stein

**Affiliations:** 1 Pediatric Pulmonology Unit, University Hospital, Federal University of Minas Gerais, Avenida Alfredo Balena 190, Room 267, Belo Horizonte, 30130-100 Brazil; 2 Municipal Public Health Department, Belo Horizonte, Brazil; 3 Laboratory of Pediatric Respirology, Infant Center, Institute of Biomedical Research, Pontifícia Universidade Católica do Rio Grande do Sul, Porto Alegre, Brazil

**Keywords:** Pediatric asthma, Asthma treatment, Asthma management

## Abstract

**Background:**

Daily inhaled corticosteroids are widely recommended for mild persistent asthma. This study aimed to assess the efficacy of the intermittent use of beclomethasone as an alternative treatment for mild persistent asthma.

**Methods:**

In this 16-week trial, children aged 6–18 years were evaluated. Subjects in the continuous treatment arm of the study received 500 μg/day of beclomethasone, whereas the intermittent ones were given 1000 μg/day (250 μg every 6 h) in combination with albuterol for 7 days upon exacerbations or worsening of symptoms. Primary outcome (i.e., treatment failure) was the occurrence of any asthma exacerbation requiring prednisone, and co-secondary outcomes were the mean/median differences for both, (1) the pre-bronchodilator FEV_1_ (% predicted) and (2) asthma control test (ACT/cACT) scores, from randomization to the last follow-up visit, and beclomethasone and albuterol consumption.

**Results:**

Ninety-four subjects from each treatment arm were included. They were comparable regarding all baseline characteristics; prednisone was used by 10 (10.6%) and 7 (7.4%) patients, respectively (95% CI − 6.1 to 12.6%, for the difference; p = 0.47). Statistical analysis showed no statistically significant differences with respect to both FEV_1_ (p = 0.39) and ACT/cACT scores (p = 0.38). As assessed through canister weighting, children used from 0.5 to 0.7 and from 1.6 to 1.8 puffs per day of beclomethasone in the intermittent and continuous regimens, respectively. Regarding albuterol, received 0.3–0.4 (intermittent) and 0.1–0.2 (continuous) inhalations per day. There were no relevant clinical or functional differences between the two treatment regimens.

**Conclusion:**

Clinicians might consider intermittent inhaled steroid therapy as a therapeutic regimen for mild persistent asthma.

*Trial registration* The Portuguese and English versions of the study protocol were submitted, approved, and registered in the Brazilian Network Platform for Clinical Trials (http://www.ensaiosclinicos.gov.br) under the primary identifier number “RBR-3gbyhk”. This platform is part of the Primary Registries in the World Health Organization Registry Network, where the trial is registered under the following Universal Trial Number: 1111-1149-4774

## Background

International guidelines consistently recommend daily use of inhaled corticosteroids (ICS) for mild persistent asthma, which accounts for the majority of persistent cases. However, three publications have evaluated the clinical benefits of an intermittent/as-needed strategy for the treatment of children with mild asthma, suggesting that although continuous treatment is associated with better disease control, the results for intermittent use were also clinically and functionally acceptable [1–3]. Studies have indicated that an on demand, intermittent regimen, combining inhaled [1, 2] or nebulized [3] corticosteroids with short acting beta 2-agonists is able to reduce the use of inhaled corticosteroids by almost 80% in these patients. In low and middle-income countries, where ICS is not widely available or affordable, reduced medication use can bring benefits for public health policies.

The present study aimed to study the efficacy of an intermittent regimen of beclomethasone dipropionate on exacerbations rate, as well as other clinical and lung function outcomes. Our hypothesis was that there would be a slight superiority in favor of the continuous regimen.

## Methods

### Study design

Two-arm, 16-week long, parallel, randomized open-label (i.e., with no placebo control) study, involving three centers. Participants were initially admitted to a 4-week run-in period, when they were prescribed two puffs (250 μg each) daily—even if they were given a lower dose of beclomethasone or equivalent previous to the run-in period—of HFA non-extrafine beclomethasone dipropionate (Clenil^®^, Chiesi, Brazil; hereafter beclomethasone), and albuterol as needed, every 4 h. Out of the two available options in Brazil, we decided to adopt the 250 μg formulation of beclomethasone instead of the 50 μg, in an attempt to reduce the number of daily puffs and achieve higher adherence rates.

In order to be included in the 16-week follow-up, patients with asthma had to be well controlled and without exacerbations during the run-in period, after which eligible patients were randomly allocated into two groups, i.e., intermittent or continuous treatment. From randomization until the end of the follow-up, patients were assessed clinically, with a complete physical examination and either the Asthma Control Test (ACT) [4] or the Childhood Asthma Control Test (cACT) [5] and pulmonary function tests. Due to the potential safety benefits regarding ICS’ side effects in the case of reduction of total daily doses, height was also systematically evaluated at every follow-up visit.

### Sampling site and randomization process

Children were recruited from the city’s public health system facilities network, where they were initially evaluated by general pediatricians and then referred to and followed by a pediatric pulmonologist along with a multi-disciplinary team allocated in three different secondary referral centers. Block randomization (30 patients per block) was used to assign participants into the two treatment regimens through a computer-generated random sequence of numbers.

### Dosage and duration of the intervention

During the 16 weeks of the follow-up, subjects assigned to the continuous group were given 500 μg daily (250 μg bid) of beclomethasone, whereas those in the intermittent group received 1000 μg daily (1 puff of 250 μg every 6 h) of beclomethasone plus 4 puffs of albuterol every 4 h for 7 days, upon the worsening of asthma symptoms and/or the onset of any asthma exacerbation.

All inhaled medications were used through a pear-shaped, plastic, large volume (650 ml) valved spacer (Flumax^®^, Inside, Brazil). Subjects were instructed to assure proper use of the spacer and inhaler devices.

### Inclusion criteria for admission into the run-in period

To ensure that only mild asthmatic patients would be recruited, we admitted participants that: had asthma symptoms but were naïve to controller treatment in the previous 2 years; had no asthma exacerbation in the previous 3 months; made regular use of inhaled corticosteroids in the previous 8 weeks (up to 500 μg daily of beclomethasone or equivalent); and their asthma was under control in the previous 8 weeks using 250–500 μg of beclomethasone (or equivalent) daily. Finally, in order to be included, children should have been able to perform spirometry and not have had smoked within the previous year.

### Exclusion criteria

In order to preclude the recruitment of patients with other severity levels, we excluded patients that had had treatment with oral steroids in the 2 weeks prior to the run-in period or within 2 weeks of screening visits; forced expiratory volume in 1 s (FEV_1_) less than 60% of predicted values [6] at the end of the run-in period; hospitalization for asthma in the previous year; presence of chronic or active disease other than asthma; asthma exacerbation in the previous 3 months or more than two in the last year; history of an exacerbation that required intensive care unit hospitalization; use of oral or injectable desensitizing immunotherapy for 3 months or more; inability to perform spirometry; and a nurse/physician impression that the family would not adhere to prescribed treatment.

### Randomization process at the end of the run-in period

Participants were selected for randomization if after the run-in period their asthma symptoms (as assessed by ACT/cACT) were controlled and FEV_1_ was equal or greater than 75% of predicted values [6].

### Definition and management of exacerbations

Exacerbations were defined as (1) the use of more than 12 puffs of albuterol daily, (2) an acute attack that led to difficulty sleeping/nighttime asthma symptoms or doing daily activities for 2 or more consecutive days, and/or (3) an unscheduled visit to one of the three secondary referral center because of worsening of asthma symptoms.

To ensure a standardized approach in both arms of the study, a written action plan form was given to patients and their parents to collect and record relevant data during the study, with particular emphasis on how to: (1) recognize asthma worsening (including difficulty sleeping/nighttime symptoms or doing daily activities for 2 or more consecutive days) and (2) manage exacerbations at home including recording the number of administered doses of albuterol and beclomethasone.

The initial home management of exacerbations should be started with the use of albuterol (3 cycles of 4–6 inhalations every 20 min and then 4 puffs every 4 h); if no improvement had been observed within the next 2–4 h, then they were instructed to add beclomethasone provisionally (1 puff of 250 μg every 6 h, i.e., 1000 μg daily). If clinical improvement or control were not achieved within 2–4 h, patients were also instructed to seek assistance by the research team at the nearest study site. The final decision to prescribe beclomethasone for up to 7 days and prednisone (1–2 mg/kg/day for 5 days) was based solely on the assessment of the pediatric pulmonologist. He also was the solely responsible for withdrawing beclomethasone among patients that needed prednisone.

### Primary and co-secondary endpoints

The primary endpoint was treatment failure, pre-defined as the occurrence of any asthma exacerbation requiring oral corticosteroid, in the two groups.

Co-secondary endpoints were also pre-defined as the mean/median difference for the pre-bronchodilator FEV_1_ (% predicted) and for ACT/cACT scoring (20 or more points means asthma control) from randomization to the last follow-up visit, and beclomethasone and albuterol consumption (expressed as number of puffs per day). Adherence rate to inhaled medicines (firstly calculated in micrograms per day and then converted to number of puffs per day) was assessed by systematic canister weighting (each actuation = 55.46 and 72.23 mg for beclomethasone and albuterol, respectively) through an analytical scale at every follow-up visit. A previous study from our group carried out in the same setting showed that the adherence assessment to beclomethasone through this method was comparable to electronic monitoring [7].

### Spirometry

Forced expiratory spirometry with recording of FEV_1_ values, was performed by an experienced technician who was blind to the treatment regimen, according to the American Thoracic Society recommendations [8]. To allow comparisons with the two previously published studies on the efficacy of on demand ICS treatment, FEV_1_ values were expressed as a percentage of the predicted value according the equations reported by Polgar and Promadhat [6].

### Height assessment

During the follow-up period, height was assessed every 2 months through a Harpenden stadiometer (ranging from 60.0 to 210.0 cm, length of 2 m, wall mounted, with a precision of 0.1 cm). Height was measured with the patient standing up, barefoot, positioned in such a way that the head, shoulders, buttocks and calves lightly touched the wall.

### Allergic rhinitis assessment and treatment

Patients with allergic rhinitis (AR) enrolled in both groups were assessed through the score reported by Wilson et al. [9] and treated with continuous intranasal budesonide (32 μg/dose, bid). Each of the typical signs and symptoms of AR received a number of points, ranging from 0 (best) to 3 (worst). The total score could range from 0 to 18 points.

### Statistics

#### Sample size

Assuming that the two regimens are in fact different, sample size calculation took into account the following parameters: (1) alpha and beta error equal to 0.05 and 0.20, respectively; (2) proportion of subjects with treatment failure of 5% (continuous arm) and 15% (intermittent arm), and a ratio of 1:1 between the two groups. A total of 282 participants were required, 141 in each group [10].

#### Analysis

Descriptive statistics was used to compare demographic, clinical and functional characteristics between the two groups. For longitudinal analysis of FEV_1_, comparisons between the two groups were done by generalized estimating equations regression model for binary response, i.e., intermittent versus continuous treatment [11]. In that case, curve fitting was plotted to demonstrate its variations during the follow-up.

Because of the specificity of its longitudinal data characteristics, comparisons of the ACT/cACT between the two groups consisted of developing linear regression with random effects model for longitudinal data, to explain the variation throughout the follow-up of each of their items [12].

The underlying assumption for these models is that the outcome is a linear function of the regression coefficients obtained for the explanatory variables.

## Results

**Fig. 1:**
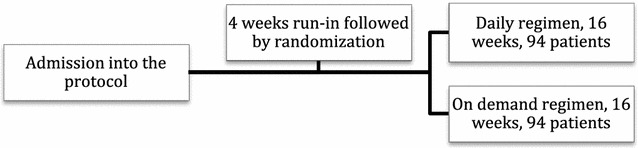
Study design

**Fig. 2:**
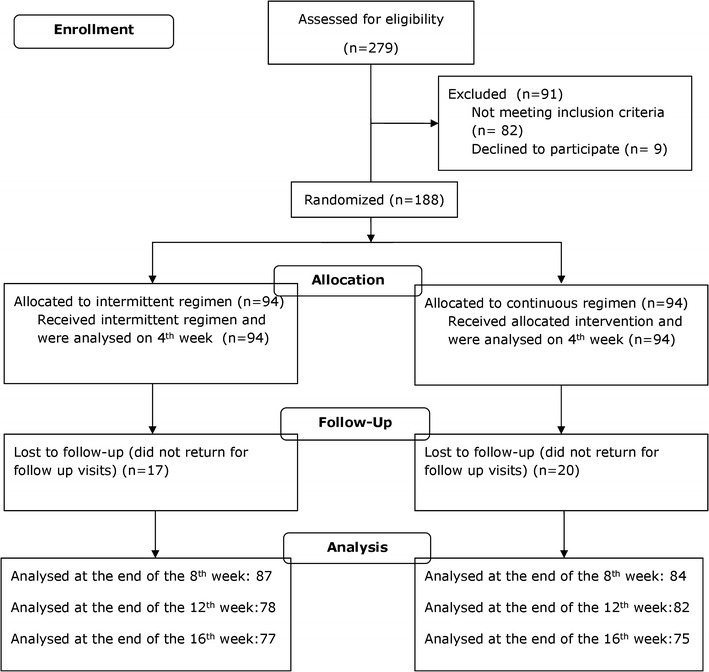
Study flow diagram

Figure 1 displays the study design and Fig. 2 the flow of the participants throughout the trial. As shown, 279 children were initially assessed for eligibility. Due to operational and funding constraints, only 188 were enrolled into the trial, and out of those, 94 were assigned to each of the two treatment regimens.

The baseline characteristics of the studied subjects are displayed on Table 1.

**Table 1:** Baseline characteristics by treatment group at randomization

	Intermittent (n = 94)	Continuous (n = 94)	p value
Age in years, mean (SD)	10.6 (2.8)	9.9 (2.7)	0.09
Sex (boys)	55 (58.5)	50 (53.2)	0.55
Height (cm)	145.1 (15.3)	142.1(14.1)	0.12
Family monthly income (Brazilian minimum wage)	2.4 (1.6)	2.3 (1.5)	0.55
Mother’s schooling (literate)	93 (99.0)	92 (97.9)	0.75
Ethnic group (white)	84 (89.3)	52 (55.3)	0.20
Number of siblings (up to three)	68 (72.3)	73 (77.6)	0.84
Parental history of allergic rhinitis (yes)	60 (63.8)	64 (68.0)	0.87
Parental history of asthma	54 (57.4)	52 (55.3)	0.81
Exposure to mould (yes)	38 (40.4)	38 (40.4)	0.88
Exposure to house dust mite (yes)	72 (76.6)	72 (76.6)	0.96
ACT/cACT score, mean (SD)	21.9 (3.0)	22.0 (2.7)	0.80
Previous ICS treatment (250–500 μg of beclomethasone or equivalent)	87 (92.5)	91 (96.8)	0.33
Allergic rhinitis/continuous intranasal budesonide (yes)	36 (38.2)	29 (30.8)	0.28
Allergic rhinitis scoring (points)^a^	6.6 (3.7)	6.1 (4.1)	0.44
Pre-bronchodilator FEV_1_ (%), mean (SD)	88.2 (12.1)	89.8 (12.0)	0.39
Bronchodilator response (%), mean (SD)	7.7 (8.5)	7.2 (7.6)	0.95

Data are proportions (%) or means (SD) unless stated otherwise; one Brazilian minimum wage corresponded approximately to US$ 250.00 during the study period

*ACT/cACT* asthma control test/childhood-asthma control test, *FEV*_*1*_ forced expiratory volume in 1 s

^a^Described by Wilson et al. [9]

The two groups were similar regarding demographic, socioeconomic, clinical, and functional characteristics.

Exacerbations were assessed at the end of follow-up and occurred in the 2nd and 3rd months of the follow-up when prednisone use was required in 10 (10.6%) and 7 (7.4%) patients of the intermittent and continuous regimen, respectively (p = 0.47). The 95% confidence interval for the difference (i.e., 3.2%) between the two (10.6 and 7.4%) proportions was − 6.1 to 12.6%.

**Fig. 3:**
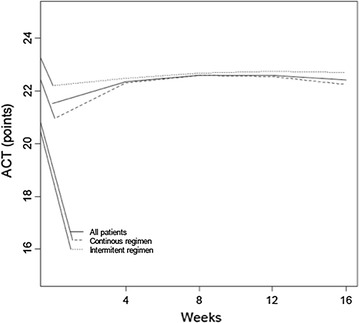
Curve fitting for ACT/cACT scores from randomization to the end of the follow up of all (continuous line), intermittent (dotted line) and continuous (dashed line) participants.

**Fig. 4:**
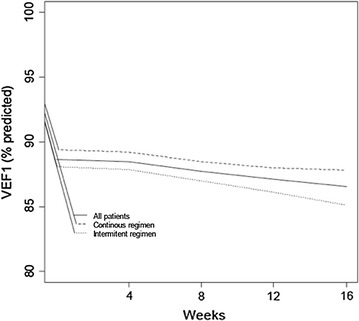
Curve fitting for FEV1 (% predicted) from randomization to the end of the follow up of all (continuous line), intermittent (dotted line) and continuous (dashed line) participants

Figures 3 and 4 show the curve fitting for FEV_1_ and ACT/cACT scores for the entire population and for the two treatment regimen groups and Table 2 the monovariate analysis from randomization to the end of the follow-up.

**Table 2:** FEV_1_ values and ACT/CACT score at randomization and at the end of the follow up by treatment group

	Intermittent			Continuous			p value
	Mean	SD	Median	Mean	SD	Median	
FEV_1_ (% predicted)							
At randomization	87.1	15.0	87.0	87.2	17.7	87.0	0.71
At the end of follow up	91.2	23.0	88.0	82.9	22.5	87.0	0.42
ACT/cACT (points)							
At randomization	21.9	3.0	23.0	22.0	2.7	23.0	0.80
At the end of follow up	22.1	3.4	24.0	21.7	3.5	23.0	0.34

Median FEV_1_ ranged from 87 to 88% of predicted values from randomization to the end of the follow-up. In the multivariate regression analysis with time (in months) and treatment regimen as covariate, only the duration of the follow-up explained FEV_1_ values, i.e., there were no statistically significant differences with respect to this functional parameter between groups (p value = 0.39). Also, they were not statistically different (p = 0.71) also for mean FEV_1_ values of 87.1% (SD 15%; median of 87%) and 87.2% (SD 17.7%; median of 87%), for intermittent and continuous regimen, respectively.

As for ACT/cACT mean and median scores, they ranged from 21 to 23 points from randomization to the end of the follow-up. Again, after multivariate regression analysis, only the duration of the follow-up determined changes in ACT/cACT (p = 0.38).

Canister weighting revealed that children allocated to the intermittent regimen used from 0.5 to 0.7 puffs/day of beclomethasone, whereas those in the continuous group used from 1.6 to 1.8 puffs/day. The overall amount of beclomethasone administered throughout the study was approximately 210 for the continuous group and 80 for the intermittent group, 60% less. Regarding the intake of albuterol, intermittent arm patients received 0.3–0.4 inhalations per day, against 0.1–0.2 for the continuous one.

As for linear growth, there was no statistically significant difference (p = 0.35) between groups. The intermittent gained 1.6 cm (SD 1.4 cm) whereas the continuous, 1.4 cm (SD 1.6 cm).

Finally, Wilson’s score of allergic rhinitis^9^ at the end of the follow-up was 5.3 (SD 3.6) for the intermittent group, and 4.8 (SD 3.4) for the continuous one. There was no statistically significant difference (p = 0.34) in comparing changes, i.e., the treatment of allergic rhinitis led to a comparable degree of control of nasal symptoms in either treatment group.

## Discussion

In the present study, we did not find any statistically significant difference in clinical and functional characteristics between the two groups. Our results suggest that the intermittent use of beclomethasone is an alternative to reduce future risk of asthma exacerbations, currently recognized as the main patient-related outcome to assess asthma control. Results also suggest that the intermittent regimen can reduce the frequency of exacerbations, even if to a lesser degree than the continuous one. Throughout the follow-up, asthma control, measured through ACT/cACT and FEV_1_, was achieved at a satisfactory level in both groups.

Our results are comparable to those observed by Turpeinen et al. [1] and Martinez et al. [2], who carried out studies with similar methodologies, but among affluent populations, and with a longer follow-up. Their main endpoints were lung function, number of exacerbations and growth. They concluded that regular use of inhaled corticosteroids led to better asthma control than the intermittent regimen. In turn, Turpeinen et al. compared the effect of inhaled budesonide given daily (59 patients) or on-demand (58 patients) for mild persistent asthma patients, aged 5–10 years [1].

Martinez et al. [2] assessed the effectiveness of beclomethasone as rescue treatment, through a placebo-controlled study where two out of four groups also received (a) twice daily beclomethasone with beclomethasone plus albuterol as rescue (71 patients), or (b) twice daily beclomethasone with placebo plus albuterol as rescue (72 patients). They concluded that, daily beclomethasone was the most effective treatment to prevent exacerbations, and ICS as rescue medication with albuterol may be an effective step-down strategy for children with well-controlled, mild asthma.

Papi et al. [3] carried out a randomized controlled trial with a similar approach. Rather than inhaled, they used nebulized beclomethasone in 276 pre-school children assigned to three groups. At the end of the 3-month follow-up, the percentage of symptom-free days was higher with regular beclomethasone (69.6%) than with prn combination (64.9%, p = 0.03). As with the two previously mentioned studies, regular ICS was the most effective treatment for frequent wheezing in preschool children, with on-demand bronchodilator/ICS combination as an alternative.

It is worth noting that in our setting, similar to other low and middle-income countries, shortage and limited access to inhaled corticosteroids brings on the need to look for strategies to optimize the required dose to maintain asthma control and avoid exacerbations.

One of our main findings was that the amount of beclomethasone used by the intermittent group was 60% that of the continuous one. As most asthmatic children requiring continuous use of inhaled corticosteroids suffer from mild persistent asthma, we can estimate that this strategy could reduce by nearly 50% the consumption of beclomethasone used by mild asthmatic patients in a given setting. In other words, as many as double the amount of patients could benefit from the intermittent regimen in low-income settings.

Adherence rate to continuous treatment regimens is a major problem in real life, and it is invariably lower than the prescribed dose because families tend to use these medications intermittently, only to resume therapy when symptoms reappear [13]. Even in more affluent societies, where at least in a part of the population, mean rates can be lower than 60% [14, 15] a rate relatively similar to the consumption of beclomethasone in our intermittent group, patients unwittingly make use of an informal intermittent therapeutic regimen. Therefore, the intermittent strategy is already part of the patients’ or their parents’ habits.

The negative impact of untreated AR on asthma control is well known, since they are pathophysiologically and clinically related. For this reason, intranasal corticosteroids do improve asthma outcomes in patients suffering from both AR and asthma. To avoid the confounder role of untreated AR among AR patients in the two arms, and differently from the previously mentioned studies [1–3], we included the diagnosis and treatment of all participants with this comorbidity to prevent the negative influence of untreated AR on the pre-defined endpoints in both arms. For instance, in a previous study carried out in the same setting, the presence of allergic rhinitis (OR 2.98, 95% CI 1.10–8.06) was an independent factor for unscheduled emergency departments visits [16]. As expected, the treatment of allergic rhinitis led to a comparable level of control of nasal symptoms in both groups and, at least theoretically, could have contributed to a better asthma control.

Our study have some limitations. The first one is related to the small population; due to financial and operational constraints, we recruited less individuals than the planned sample size. The fact that there was no difference in exacerbation rates could have been caused by lack of power. Secondly, a 16-week follow-up is suboptimal for a study assessing asthma exacerbations as primary outcome. Thus, a longer follow-up would be ideal, as seen in Martinez et al. [2] and Turpeinen et al. [1]. In the Turpeinen study [1], a difference in exacerbation rates was only noted during the second year of the treatment strategy. Likewise, the lack of growth effects and further increases in ACT/cACT and FEV_1_ values might also be due to lack of power. All in all, the trial is underpowered, and no firm conclusions can be made.

However, as the 95% CI for the difference (3.2%) between the two groups, shows a range that might favor both regimens, we could speculate that adding more patients would not make that much of a difference. It is reasonable to presume that at least one exacerbation may occur within 16 weeks among patients suffering from undertreated/uncontrolled mild persistent asthma. The shape of the longitudinal curves for FEV_1_ and ACT/cACT showed a homogenous pattern throughout the follow-up, demonstrating a comparable level of clinical and functional control in the two treatment groups.

## Conclusion

Our results are in line and confirm those obtained in the three previously mentioned works and, therefore suggest that the intermittent strategy might be used as an alternative regimen for children suffering from mild persistent asthma.

Our study was conducted in a real life primary and secondary public health system facilities network with children from low-income families, which is more likely to increase the clinical applicability of our findings. As pointed out by Martinez et al. [2], this approach might also be an alternative for stepping down beclomethasone after asthma control is achieved among patients suffering from mild persistent asthma especially in poor resource settings.

Finally, clinicians/pediatricians should carefully weigh the potential advantages and disadvantages of each of the two treatment strategies in individualized assessments and possibly in the context of a shared decision-making [17].

## Abbreviations

ICS: inhaled corticosteroids
ACT: asthma control test
cACT: childhood asthma control test
FEV_1_: forced expiratory volume in 1 s
AR: allergic rhinitis
SD: standard deviation
